# Commonalities in EEG Spectral Power Abnormalities Between Women With ADHD and Women With Bipolar Disorder During Rest and Cognitive Performance

**DOI:** 10.1007/s10548-016-0508-0

**Published:** 2016-07-27

**Authors:** Anna-Sophie Rommel, Glenn L. Kitsune, Giorgia Michelini, Georgina M. Hosang, Philip Asherson, Gráinne McLoughlin, Daniel Brandeis, Jonna Kuntsi

**Affiliations:** 1 King’s College London, MRC Social, Genetic and Developmental Psychiatry Centre, Institute of Psychiatry, Psychology and Neuroscience, London, UK; 2 King’s College London, Department of Psychological Medicine, Institute of Psychiatry, Psychology and Neuroscience, London, UK; 3 Department of Psychology, Goldsmiths University of London, London, UK; 4 Department of Child and Adolescent Psychiatry and Psychotherapy, Central Institute of Mental Health, Medical Faculty Mannheim, Heidelberg University, Mannheim, Germany; 5 Department of Child and Adolescent Psychiatry and Psychotherapy, Psychiatric Hospital, University of Zurich, Zurich, Switzerland; 6 Center for Integrative Human Physiology, University of Zurich, Zurich, Switzerland; 7 Neuroscience Center Zurich, University of Zurich, Zurich, Switzerland

**Keywords:** ADHD, Bipolar disorder, Quantitative EEG, Spectral power, Theta power

## Abstract

While attention-deficit/hyperactivity disorder (ADHD) and bipolar disorder (BD) denote distinct psychiatric conditions, diagnostic delineation is impeded by considerable symptomatic overlap. Direct comparisons across ADHD and BD on neurophysiological measures are limited. They could inform us on impairments that are specific to or shared between the disorders and, therefore, potential biomarkers that may aid in the identification of the diagnostic boundaries. Our aim was to test whether quantitative EEG (QEEG) identifies differences or similarities between women with ADHD and women with BD during resting-state and task conditions. QEEG activity was directly compared between 20 ADHD, 20 BD and 20 control women during an eyes-open resting-state condition (EO) and a cued continuous performance task (CPT-OX). Both ADHD (t_38_ = 2.50, p = 0.017) and BD (t_38_ = 2.54, p = 0.018) participants showed higher absolute theta power during EO than controls. No significant differences emerged between the two clinical groups. While control participants showed a task-related increase in absolute theta power from EO to CPT-OX (t_19_ = −3.77, p = 0.001), no such change in absolute theta power was observed in the ADHD (t_19_ = −0.605, p = 0.553) or BD (t_19_ = 1.82, p = 0.084) groups. Our results provide evidence for commonalities in brain dysfunction between ADHD and BD. Absolute theta power may play a role as a marker of neurobiological processes in both disorders.

**Electronic supplementary material:**

The online version of this article (doi:10.1007/s10548-016-0508-0) contains supplementary material, which is available to authorized users.

## Introduction

Attention-deficit/hyperactivity disorder (ADHD) and bipolar disorder (BD) are common psychiatric disorders, respectively affecting around 2–4 % and 1–2 % of the adult population worldwide (Merikangas et al. 2011; Willcutt 2012). While ADHD and BD denote distinct psychiatric conditions (American Psychiatric Association 2013), diagnostic delineation is impeded by considerable symptomatic overlap. Both ADHD and the manic phase of BD are associated with distractibility, restlessness, talkativeness and lack of social inhibition (Kent and Craddock 2003; Galanter and Leibenluft 2008). Both disorders further present with features of mood dysregulation, such as irritability and emotional lability (Skirrow et al. 2012, 2014). However, ADHD symptoms are chronic and trait-like, while BD symptoms tend to occur for distinct periods of time (Asherson et al. 2014). Nevertheless, symptoms of distractibility and mood dysregulation (Najt et al. 2007; Peluso et al. 2007; Newman and Meyer 2014), as well as residual cognitive and functional impairments (Torres et al. 2007; Henry et al. 2013), persist as milder stable traits in euthymic BD. Such overlap can lead to challenges in distinguishing the two disorders, or recognising comorbidity, in clinical practice and may consequently result in inappropriate treatment decisions (Asherson et al. 2014).

Similar cognitive impairments have also been described for individuals with ADHD and BD. Both ADHD and euthymic BD are associated with poor accuracy in attentional and inhibitory processing tasks (Robinson and Ferrier 2006; Arts et al. 2008; McLoughlin et al. 2010; Torralva et al. 2011), as well as increased reaction time variability (RTV) (Brotman et al. 2009; Kuntsi et al. 2010; Kuntsi and Klein 2012; Adleman et al. 2014). Yet, similar cognitive performance could stem from differing underlying mechanisms (Banaschewski and Brandeis 2007). Consequently, our recent cognitive-electrophysiological investigations of attentional and inhibitory processing in women with ADHD and women with BD revealed evidence for disorder-specific impairments, despite indistinguishable cognitive performance (Michelini et al. 2016). Event-related potential (ERP) analyses showed a significantly reduced N2 amplitude in participants with BD, compared to the ADHD and control groups, in response to NoGo stimuli during a cued continuous performance task (CPT) (Michelini et al. 2016). As the N2 in response to NoGo stimuli or in incongruent trials is considered to reflect conflict-monitoring processing (Yeung and Cohen 2006), the results suggest impaired conflict monitoring in women with BD, compared to women with ADHD and control women. Yet, women with ADHD and women with BD also showed overlapping neurophysiological impairments compared to controls in the NoGo-P3, suggesting shared inhibitory control deficits (Michelini et al. 2016).

Another method to investigate covert processing and other underlying mechanisms in the absence of overt performance differences is employing quantitative electroencephalography (QEEG). QEEG allows the direct examination of subtle changes in cortical activity which may reflect state regulation and arousal (Banaschewski and Brandeis 2007). This is of particular relevance in conditions such as ADHD and BD which show abnormalities in state regulation and arousal (Degabriele and Lagopoulos 2009; Ongür et al. 2010; Cortese et al. 2012; Nigg 2013). In QEEG, electrophysiological recordings are quantified in the frequency ranges delta (0.5–3.5 Hz), theta (3.5–7.5 Hz), alpha (7.5–12.5 Hz), beta (12.5–30 HZ) and gamma (>30 Hz). The most consistently reported findings of QEEG studies in children and adults with ADHD during resting-state conditions are elevated power in slow (delta and theta) frequency bands, reduced power in fast wave cortical activity (mainly beta) and an elevated proportion of slower to faster frequencies in the brain, as reflected in theta/beta ratio (TBR), particularly apparent at fronto-central sites (Bresnahan et al. 1999; Bresnahan and Barry 2002; Clarke et al. 2003, 2006; Snyder and Hall 2006; Clarke et al. 2008; Koehler et al. 2009; Cooper et al. 2014). This has also been confirmed by meta-analyses, reporting effect sizes between 0.58 and 1.31 for theta power and between 0.62 and 3.08 for TBR (Boutros et al. 2005; Snyder and Hall 2006; Arns et al. 2013). Yet, several recent studies have failed to replicate these findings (Loo et al. 2009; Ogrim et al. 2012; Liechti et al. 2013; Buyck and Wiersema 2014; Poil et al. 2014; Kitsune et al. 2015; Skirrow et al. 2015) and the increased TBR as a marker of ADHD diagnosis is being contested (Arns et al. 2013; Lenartowicz and Loo 2014; Jeste et al. 2015; Arns et al. 2016). EEG spectral power in ADHD further seems to depend on the context, with one study finding elevated delta and theta activity in individuals with ADHD compared to controls during the resting-state condition at the start of recording sessions and increased beta power only at the end of the recording session in ADHD (Kitsune et al. 2015). In BD, elevated delta and theta power, as well as decreases in alpha power, during resting-state conditions have been reported (Clementz et al. 1994; Degabriele and Lagopoulos 2009; Başar et al. 2012). However, direct EEG comparison studies between ADHD and BD have not yet been conducted.

Few studies on ADHD have examined cortical activity patterns during cognitive task conditions and findings are inconsistent. While some studies have shown no differences in cortical activation between controls and individuals with ADHD during a CPT (Loo et al. 2009; Skirrow et al. 2015), others have reported elevated alpha (Swartwood et al. 2003; Nazari et al. 2011) and theta (El-Sayed et al. 2002) power in individuals with ADHD compared to controls. In addition, lower theta power in adults with ADHD has been demonstrated in the sustained attention to response task (SART), owing to task-related increase in frontal theta activity in control participants that was absent in participants with ADHD (Skirrow et al. 2015). Treatment with methylphenidate resulted in normalisation of the resting-state to task activation pattern. These findings may indicate a lack of modulation of cortical activity from resting-state to cognitive task in the ADHD group compared to controls. QEEG profiles of individuals with BD during cognitive tasks have not yet been studied. Investigating the oscillatory patters of individuals with ADHD and BD across conditions, from rest to cognitive task condition, may allow us to investigate cortical activation and arousal patterns that could inform us on impairments that are specific to or shared between the disorders.

The aim of this study was to test whether quantitative EEG identifies differences or similarities between women with ADHD, women with bipolar disorder and controls during a resting-state condition (eyes open) and an active task condition (a flanked continuous performance test), which could inform us on overlapping and distinct electrophysiological impairments in both disorders that may underlie symptomatic and cognitive similarities.

## Method

### Sample

The sample consisted of 20 women with ADHD, 20 women with euthymic BD and 20 control women. Participants with ADHD were recruited from the Adult ADHD Clinic at the Maudsley Hospital, London, UK. Participants with BD were recruited from the Maudsley Psychosis Clinic, London, UK, or had previously participated in another research study (Hosang et al. 2012). Control participants were recruited from the Mindsearch volunteer database maintained by the Institute of Psychiatry, Psychology and Neuroscience, King’s College London, UK, which comprises several thousand potential participants. Participants for this study were randomly selected from all those meeting inclusion criteria.

Diagnosis in the clinical groups was first assessed with the help of medical records, following Diagnostic and Statistical Manual (DSM-IV) criteria (American Psychiatric Association 2000) and later confirmed during the research assessment using the Diagnostic Interview for Adult ADHD (DIVA, Kooij and Francken 2007), the Altman Self-Rating Mania Scale (Altman et al. 1997), the Becks Depression Inventory (Beck et al. 1996), as well as the Young Mania Rating Scale (Young et al. 1978). The ADHD participants met current criteria for combined-type ADHD or inattentive-type ADHD with sufficient symptoms of hyperactivity-impulsivity in the past to meet a childhood combined-type diagnosis. Participants in the BD group had a diagnosis of bipolar I disorder (BD-I), with evidence of a past manic episode lasting 1 week or more. BD-I patients were selected if they were currently euthymic, meaning that they were not experiencing a manic or depressed episode at the time of the assessment. Exclusion criteria for all groups were drug or alcohol dependency in the last 6 months, autism, epilepsy, neurological disorders, brain injury, past ECT treatment, current involvement in another research trial likely to alter symptom severity, pregnancy or a limited proficiency in English language. Those with a comorbidity of both ADHD and BD, or who were currently experiencing a manic episode, were also excluded. In addition, control participants, who reported a history of psychiatric disorders or who were taking psychiatric medication, were excluded from the study.

All participants had normal or corrected-to-normal vision. Participants’ IQs were assessed with the Wechsler Abbreviated Scale of Intelligence–Fourth Edition (WASI-IV; Wechsler 1999). IQ (F_2,58_ = 1.37, p = 0.26) and age (F_2,59_ = 1.63, p = 0.21), which ranged from 20 to 52 years, did not differ between groups (Table 1). Participants with ADHD were asked to come off stimulant medication 48 h before the assessment. For ethical reasons, participants were not asked to stop taking mood stabilisers (70 % of the BD group), anti-psychotic medication (40 % of the BD group) or anti-depressants (7 % of the ADHD group and 25 % of the BD group) they had been prescribed. All participants were asked to refrain from caffeinated drinks and nicotine 2 h prior to the testing session. The investigation was carried out in accordance with the latest version of the Declaration of Helsinki. Ethical approval for the study was granted by the Camberwell St Giles Research Ethics Committee (approval number 11/LO/0438) and all participants provided after the nature of the procedures had been fully explained.

**Table 1:** Demographic data: mean (SD) and p-value from ANOVA

	ADHD	BD	Controls	p-value
Age (years)	37.4 (7.6)	40.3 (7.7)	36.7 (4.3)	0.21
IQ	104.5 (17.9)	108.0 (12.5)	112.4 (14.2)	0.26

### Procedure and Cognitive-Performance Measures

Participants completed the cognitive-EEG assessment, including an IQ test and clinical interviews, in a single 4.5 h research session. Participants completed a 3-minute eyes-open resting-state condition (EO) as well as a 3-minutes eyes-closed (EC) resting-state condition prior to performing on a CPT with flankers (CPT-OX) (McLoughlin et al. 2010; Doehnert et al. 2010; McLoughlin et al. 2011). QEEG differences between EO and CPT-OX are analysed here, in line with recent research (Nazari et al. 2011; Skirrow et al. 2015), since EO has been suggested to provide a more appropriate baseline than EC for tasks involving visual processing (Barry et al. 2007).

The CPT-OX is a cued-Go/NoGo task that probes attention, preparation and response inhibition. The task consisted of 400 black letter arrays, made up of a centre letter and incompatible flankers on each side to increase difficulty for adults. The presented arrays included the cue letter ‘O’, the target letter ‘X’ as well as the distractors ‘H’, ‘B’, ‘C’, ‘D’, ‘E’, ‘F’, ‘G’, ‘J’ and ‘L’. Letters were presented centrally on the computer monitor, subtending approximately 5°. Cue and target letters (‘O’ and ‘X’ respectively) were flanked by the incompatible letters (‘XOX’ and ‘OXO’ respectively). Participants were instructed to ignore the flanking letters and respond as quickly as possible to cue-target sequences (‘O’-‘X’). 80 Cues (‘XOX’) were followed by the target (‘OXO’) in 40 trials (Go condition), and by neutral distractors in the remainder of trials (NoGo condition). On 40 trials, the target letter ‘X’ was not preceded by a cue ‘O’ and had to be ignored. Letters were presented every 1.65 s for 150 ms in a pseudo-randomised order. Ten practice trials preceded the main task and were repeated, if required, to ensure participant comprehension. Participants were instructed to respond only to Cue-Go sequences by pressing a button as quickly as possible with the index finger of their preferred hand. Participants were further asked to withhold the response in the presence of a NoGo stimulus, in the presence of a Go stimulus not preceded by a Cue, or in the presence of any other irrelevant letters. Task duration was 11 min.

### Electrophysiological Recording and Analysis

The EEG was recorded from a 62 channel direct-current-coupled recording system (extended 10–20 montage), using a 500 Hz sampling-rate and impedances under 10 kΩ. FCz and AFz were the recording reference and ground electrodes, respectively. The electro-oculograms were recorded from electrodes above and below the left eye and at the outer canthi. Participants were seated on a height-adjustable chair in a dimly lit video-monitored testing cubicle. Stimuli were presented on a computer monitor at a distance of approximately 120 cm, using the Presentation software package (www.neurobs.com). EEG data were analysed using Brain Vision Analyzer 2.0 (Brain Products, Germany). Researchers were blind to group status during EEG pre-processing and analysis. Raw EEG recordings were down-sampled to 256 Hz, re-referenced to the average of all electrodes, and digitally filtered using Butterworth band-pass filters (0.1–30 Hz, 24 dB/oct). All trials were also visually inspected for electrical artefacts (due to electrical noise in the EEG recording) or obvious movement, and sections of data containing artefacts were removed manually. Ocular artefacts, corresponding to blink-related and vertical and horizontal eye movements, were identified using the infomax independent component analysis (ICA) algorithm (Jung et al. 2000) in segmented data. The ICA algorithm (Jung et al. 2000) allows for removal of activity associated with ocular artefacts by back-projection of all but this activity. The mean number (and standard deviation) of independent components removed in the ADHD, BD and control groups respectively were 3.55 (1.23), 3.65 (1.81) and 3.20 (1.40) during EO; and 2.35 (0.67), 2.50 (0.76) and 2.45 (2.05) during CPT-OX. Sections of data with remaining artefacts exceeding ± 100 μV in any channel or with a voltage step greater than 50 μV were automatically rejected.

Quantitative EEG was investigated for EO and CPT-OX. Artefact-free data were segmented into 2-second epochs and power spectra were computed using a Fast Fourier Transform (FFT) with a 10 % Hanning window. The mean duration (and standard deviation) of the segmented data in the ADHD, BD and control groups respectively were 2.90 min (0.22), 2.93 min (0.23) and 2.95 (0.20) during EO; and 7.91 min (1.65), 8.41 min (1.51) and 8.30 min (1.10) during CPT-OX. In order to examine specific aspects of stimulus–response processing, CPT-OX data were also segmented into stimulus-locked epochs (stimulus window from 0 to 1400 ms) based on three different response conditions: Cue, Go and NoGo. Only trials with correct responses (Go) or correctly rejected trials (NoGo and Cue), and which contained at least 20 artefact-free segments, were included.

**Fig. 1:**
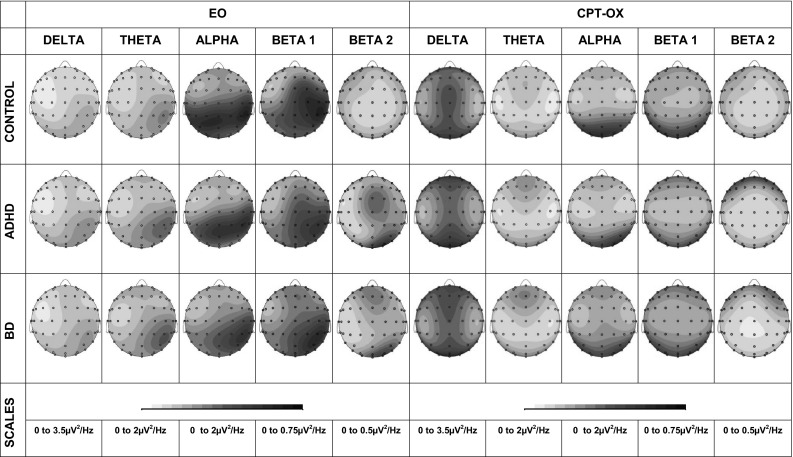
Topographic maps showing *scalp* recorded power density in absolute delta, theta, alpha, beta 1 and beta 2 bands for resting-state (EO) and CPT-OX conditions

Analyses focused on absolute delta (0.5–3.5 Hz), theta (3.5–7.5 Hz), alpha (7.5–12.5 Hz), beta 1 (12.5–18.5 Hz) and beta 2 (18.5–30 Hz) frequency band differences, as well as differences in theta/beta ratio (TBR), between ADHD, BD and control groups. All data were natural-log transformed (ln) to normalise the data. The normal distribution of log-transformed data was confirmed using a Shapiro–Wilk test. In line with previous studies (Loo et al. 2009; Skirrow et al. 2015), absolute EEG power (μV^2^) within each frequency band was averaged across frontal (Fz, F1, F2, F3, F4, F5, F6, F7, F8), central (Cz, C1, C2, C3, C4, C5, C6) and parietal (Pz, P3, P4, P7, P8) regions from individual scalp electrodes to reduce the number of statistical comparisons (see Fig. 1 for topographic maps showing scalp-recorded power density in delta, theta, alpha, beta 1 and beta 2 bands). Results for relative EEG power (μV^2^) within each frequency band can be found in the supplementary material (S1).

### Statistical Analysis

A repeated-measures analysis of variance (ANOVA), applying a Greenhouse-Geiser correction when appropriate, was carried out to investigate diagnostic status-related differences between ADHD, BD and controls in EEG power. Recording condition (EO, CPT-OX) and recording site (frontal, central, parietal) were used as within-subject variables and diagnostic status (ADHD, BD, control) as a between-subjects variable. Delta, theta, alpha, beta 1 and beta 2 power were each investigated with a 2 × 3 × 3 repeated measures ANOVA. Post-hoc analyses were carried out using independent samples *t* tests for between-subjects contrasts, and paired samples *t* tests for within-individual task related differences in EEG power. Effect sizes (Cohen’s d), which were calculated using the difference in the means divided by the pooled standard deviation (Cohen 1988), are reported. According to Cohen (1988), d = 0.20 constitutes a small effect, d = 0.50 a medium effect and d = 0.80 a large effect.

## Results

### Absolute EEG Power

The repeated-measure ANOVA indicated no significant main effects of group for absolute delta (F_2,57_ = 1.29, p = 0.283), theta (F_2,57_ = 1.70, p = 0.193), alpha (F_2,57_ = 1.20, p = 0.312), beta 1 (F_2,57_ = 0.51, p = 0.602) and beta 2 (F_2,57_ = 0.29, p = 0.747) power.

Significant main effects of recording site were identified for absolute delta (F_1,57_ = 684.90, p < 0.001; Greenhouse-Geisser ε = 0.615), theta (F_1,57_ = 140.43, p < 0.001; Greenhouse-Geisser ε = 0.871), alpha (F_1,57_ = 232.83, p < 0.001; Greenhouse-Geisser ε = 0.858), beta 1 (F_1,57_ = 89.63, p < 0.001; Greenhouse-Geisser ε = 0.802) and beta 2 (F_1,57_ = 5.81, p = 0.008; Greenhouse-Geisser ε = 0.776) power.

There were significant main effects of testing condition for absolute delta (F_1,57_ = 170.87, p < 0.01), beta 1 (F_1,57_ = 39.30, p < 0.01) and beta 2 (F_1,57_ = 19.79 p < 0.01) power, but not for absolute theta (F_1,57_ = 2.09, p = 0.154) and alpha (F_1,57_ = 3.83, p = 0.055) power.

No significant group-by-condition interaction emerged for absolute delta (F_1,57_ = 2.98, p = 0.059), alpha (F_1,57_ = 1.87, p = 0.163), beta 1 (F_1,57_ = 0.32, p = 0.728) or beta 2 (F_1,57_ = 0.99, p = 0.377) power. Consequently, the results for these frequency bands are not reported further.

**Fig. 2:**
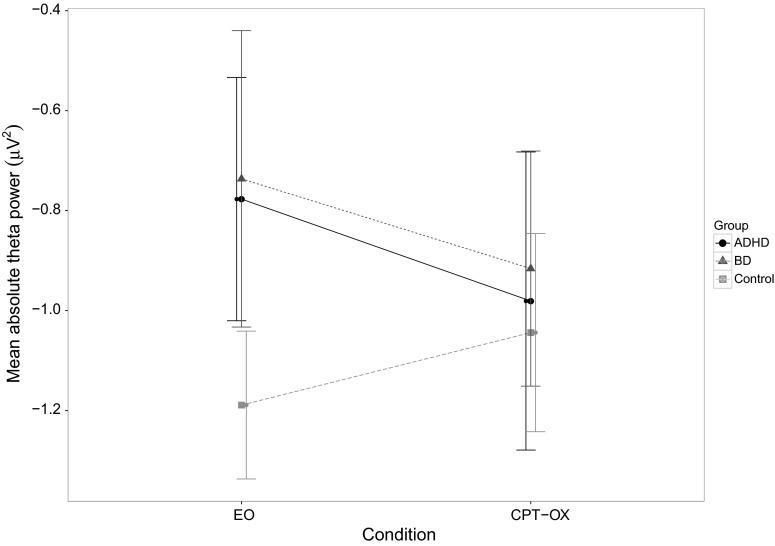
Mean absolute theta power across resting-state (EO) and task (CPT-OX) condition in the bipolar disorder (*dotted line with triangular marker*), ADHD (*solid line with round marker*) and control groups (*dashed line with square marker*). *Error bars* represent 95 % confidence intervals

A significant group-by-condition interaction, with a moderate effect size, emerged for absolute theta power (F_1,57_ = 3.39, p = 0.041, η^2^ = 0.106). Post-hoc tests revealed significantly higher absolute theta power in the ADHD group compared to controls during the resting-state condition (t_38_ = 2.45, p = 0.019), with moderate-to-large effect size (d = 0.77), but not during CPT-OX (t_38_ = 0.07, p = 0.943, d = 0.02), as well as significantly higher absolute theta power in the BD group compared to controls during the resting-state condition (t_38_ = 2.39, p = 0.022), with moderate-to-large effect size (d = 0.76), but not during CPT-OX (t_38_ = 0.80, p = 0.428, d = 0.25). Post-hoc tests showed no significant differences in absolute theta power between the ADHD and BD groups during the resting-state condition (t_38_ = 0.21, p = 0.837, d = 0.07) or during CPT-OX (t_38_ = 0.59, p = 0.561, d = 0.19). While control participants showed a task-related increase in absolute theta power (t_19_ = 3.34, p = 0.003), no significant changes in absolute theta power from EO to CPT-OX were observed in the ADHD (t_19_ = −1.23, p = 0.235) or BD (t_19_ = −1.50, p = 0.150) groups (Fig. 2). This change in absolute theta power in the control participants likely drives the significant group-by-condition interaction.

When CPT-OX was segmented based on stimulus-locked epochs (Cue, Go and NoGo), no significant group-by-condition interaction emerged for absolute delta (F_1,57_ = 2.81, p = 0.061, Greenhouse-Geisser ε = 0.500), alpha (F_1,57_ = 2.18, p = 0.114, Greenhouse-Geisser ε = 0.383), beta 1 (F_1,57_ = 2.68, p = 0.068, Greenhouse-Geisser ε = 0.390) or beta 2 (F_1,57_ = 2.43, p = 0.078, Greenhouse-Geisser ε = 0.384) power. A significant group-by-condition interaction, with a moderate effect size, emerged for absolute theta power (F_1,57_ = 3.21, p = 0.019, η^2^ = 0.101, Greenhouse-Geisser ε = 0.598) when CPT-OX was segmented based on stimulus-locked epochs (Cue, Go and NoGo).

Post-hoc tests revealed significantly higher absolute theta power in the ADHD group, compared to controls, during the resting-state condition (t_38_ = 2.50, p = 0.017, d = 0.77), but not during the Cue (t_38_ = −1.01, p = 0.317), Go (t_38_ = −1.05, p = 0.302) and NoGo (t_38_ = −0.82, p = 0.417) conditions. Post-hoc tests also demonstrated significantly higher absolute theta power in the BD group compared to controls during the resting-state condition (t_38_ = 2.54, p = 0.016, d = 0.76), but not during the Cue (t_38_ = 0.07, p = 0.948), Go (t_38_ = 0.47, p = 0.640) and NoGo (t_38_ = 0.24, p = 0.813) conditions. No significant differences in absolute theta power emerged between the ADHD and BD groups during the resting-state condition (t_38_ = 0.32, p = 0.748), Cue (t_38_ = −1.01, p = 0.318), Go (t_38_ = −1.42, p = 0.164) or NoGo (t_38_ = −1.00, p = 0.323) conditions.

### Theta/Beta Ratio (TBR)

No significant main effect of group (F_1,57_ = 1.86, p = 0.165), condition (F_1,57_ = 1.44, p = 0.706) or site (F_1,57_ = 1.43, p = 0.240) and no significant group-by-condition interaction emerged for TBR (F_1,57_ = 0.70, p = 0.503).

## Discussion

In this study investigating the relationship of EEG indices of cortical activity in women with ADHD, women with BD and control women, both ADHD and BD participants showed higher absolute theta power than controls during the resting-state condition. No significant differences emerged between the two clinical groups. While control participants showed a task-related increase in absolute theta activity from resting-state to cognitive task, no significant changes in absolute theta power were observed in the ADHD or BD groups. Our results provide evidence for commonalities in brain dysfunction between ADHD and BD. Absolute theta power may act as a marker of neurobiological processes in both disorders.

Both the ADHD and BD groups showed an elevation of absolute theta power during the resting-state condition, compared to controls. To date, no study has directly compared the cortical activity patterns of individuals with ADHD and BD. This finding suggests commonalities in oscillation patters between women with ADHD and BD. The lack of significant differences between the clinical groups adds to previous research, which has shown an elevation of theta power during resting-state conditions independently in individuals with ADHD (Bresnahan et al. 1999; Bresnahan and Barry 2002; Clarke et al. 2003, 2006; Snyder and Hall 2006; Clarke et al. 2008; Koehler et al. 2009) and in individuals with BD (Degabriele and Lagopoulos 2009), compared to controls. It is not fully understood what increased theta power in individuals with ADHD and BD during resting-state conditions represents. The findings of elevated resting theta power in younger compared to older neurotypical children (Benninger et al. 1984; Gasser et al. 1988a, 1988b) led to the development of a maturational-lag hypothesis (Kinsbourne 1973). This hypothesis holds that that there is a delay in central nervous system (CNS) development in individuals with ADHD because during neurotypical CNS maturation slow wave activity is replaced with fast wave activity. Yet, our and other research demonstrating elevated theta power in adolescents and adults with ADHD (Bresnahan et al. 1999; Bresnahan and Barry 2002; Clarke et al. 2008; Koehler et al. 2009; Kitsune et al. 2015; Skirrow et al. 2015) and BD (Degabriele and Lagopoulos 2009) do not support this hypothesis. Increased theta power in individuals with ADHD during resting-state conditions has also been interpreted as representing hypo-arousal (Satterfield and Dawson 1971; Lubar 1991). Yet, two studies investigating the relationship between resting EEG power and skin conductance level (a traditional marker of CNS arousal) in children with and without ADHD linked increased alpha rather than theta to under-arousal as indexed by skin conductance level (Barry et al. 2004, 2009). While the significance of increased theta power during resting-state conditions remains to be fully elucidated, our findings may suggest a role for absolute theta power as a common marker of neurobiological processes in both ADHD and BD. This is in line with findings from quantitative genetics studies, which have found strong phenotypic and genetic links between ADHD and abnormal theta activity, suggesting it may be a biological marker or intermediate phenotype (endophenotype) for ADHD (McLoughlin et al. 2014; Tye et al. 2014).

In addition, no differences in EEG power were observed between the three groups during the cognitive task condition, even when specific aspects of stimulus and response processing were investigated separately, and no change in absolute theta power from resting-state to task condition in the clinical groups was found. Our study is the first to investigate the QEEG profile of individuals with BD during a cognitive task and to directly compare it to an ADHD group. The findings, therefore, suggest commonalities in brain dysfunction between ADHD and BD during this cognitive task. Furthermore, this study is the first to investigate the EEG patterns during both rest and task condition in women with adult ADHD. The results support our previous work in an all-male sample, which showed no differences in cortical activation between controls and individuals with ADHD during the CPT and no change in spectral power from resting-state to cognitive task (Skirrow et al. 2015); although, previous QEEG studies have yielded inconsistent results such as elevated alpha (Swartwood et al. 2003; Nazari et al. 2011) and theta (El-Sayed et al. 2002) power on switching to CPT from resting-state in individuals with ADHD compared to controls. The seeming lack of task-dependent modulation of absolute theta power in ADHD and BD participants may potentially be explained by abnormalities in the default mode network (DMN), which is typically activated during resting-state conditions and deactivated during task performance (Broyd et al. 2009; Raichle 2010). Abnormalities in the DMN during rest have been demonstrated for both ADHD and BD (Ongür et al. 2010; Cortese et al. 2012). Yet, while task-related modulation remains to be examined in BD, the DMN has been found to be inadequately attenuated when individuals with ADHD perform a task (Sonuga-Barke and Castellanos 2007; Fassbender et al. 2009; Cortese et al. 2012). The absence of task-related changes in absolute theta power in our sample of women with ADHD and BD, as well as in previous research on ADHD (Skirrow et al. 2015), might therefore indicate inadequate attenuation of the DMN. A recent review, summarising findings from studies employing functional magnetic resonance imaging and EEG simultaneously, provides support for this idea (Nishida et al. 2015), by concluding that increased theta power indexes decreased DMN activity. Consequently, theta power may be vital to the attenuating processes required for cognitive functioning.

Unlike previous research, this study did not find elevated delta power in individuals with BD (Degabriele and Lagopoulos 2009) or decreased beta activity and an increased theta/beta ratio in individuals with ADHD (Bresnahan et al. 1999; Bresnahan and Barry 2002; Clarke et al. 2008; Koehler et al. 2009). These discrepancies may be due to age and gender effects. Our all-female sample had a mean age of 38 years and an age range of 20–52 years. As EEG power tends to decline with age (Lüchinger et al. 2011; Michels et al. 2013; Poil et al. 2014), this wide age range may have reduced power to detect differences of smaller effect between the groups. Yet, some recent studies have also failed to replicate previous findings of decreased beta power and an increased theta/beta ratio in individuals with ADHD (Loo et al. 2009; Ogrim et al. 2012; Liechti et al. 2013; Buyck and Wiersema 2014; Poil et al. 2014; Skirrow et al. 2015) and the importance of an increased TBR as a marker of ADHD is being contested (Arns et al. 2013; Lenartowicz and Loo 2014; Jeste et al. 2015; Arns et al. 2016). A recent meta-analysis demonstrated that the reported effect size for TBR abnormalities in ADHD showed a strong relationship with year of publication, declining over time (Arns et al. 2013). The paper proposes the trend for reduced sleep duration in children across time, as well as sample and testing context differences between studies as possible explanations. Support for context effects comes from a study of resting-state EEG power differences between recordings made at the beginning and the end of a 1.5 h testing session in 76 adolescents and young adults with ADHD and 85 controls, which showed elevated delta and theta power in the ADHD group in the beginning and elevated beta power in the ADHD group at the end of the testing session (Kitsune et al. 2015).

Several limitations should be considered alongside these results. Firstly, while participants were asked to come off stimulant medication 48 h before the assessment, participants were not asked to discontinue mood-stabilising, anti-psychotic or anti-depressant medication for ethical reasons. Although the understanding of the effects of medications on QEEG is still limited, no significant differences between medicated and unmedicated individuals with euthymic BD on QEEG have been found (El-Badri et al. 2001; Degabriele and Lagopoulos 2009). It is, therefore, unlikely that the results in this study were produced by medication effects. Secondly, this investigation was conducted in a homogenous all-female sample. Our results support previous work in an all-male sample, which showed no differences in cortical activation between controls and individuals with ADHD during the CPT and no change in spectral power from EO to CPT (Skirrow et al. 2015). Nevertheless, future studies are needed to replicate these findings in more typical adult ADHD and BD populations with approximately equal distribution of males and females (Biederman et al. 2004; Ayuso-Mateos 2006; Rucklidge 2010). Finally, two experimental conditions with different durations (3 min in EO and 11 min in CPT-OX) were compared in this study. It is possible that these discrepant experimental timings might have affected the result. Yet, segmenting the CPT-OX based on stimulus-locked epochs (Cue, Go and NoGo) resulted in similar findings, suggesting that the duration of the two experimental conditions is unlikely to have an impact on the results.

Our results provide evidence for commonalities in brain dysfunction between ADHD and BD, with absolute theta power potentially playing a role as a marker of shared neurobiological processes in both disorders. In light of shared cognitive impairments and the overlapping symptomatology of ADHD and BD, these findings represent a move towards uncovering biological markers underlying the pathophysiology shared between the disorders. Currently, diagnostic manuals such as the DSM (American Psychiatric Association 2000, 2013) outline clinical diagnoses in a categorical system based on the description of behavioural symptoms. Yet, research has revealed substantial evidence for pathophysiological heterogeneity within disorders (Sjöwall et al. 2013; Burdick et al. 2015; Jeste et al. 2015), as well as pathogenic overlap between disorders (Lee et al. 2013; Michelini et al. 2016). Consequently, diagnostic boundaries based on behavioural symptoms do not seem to correspond seamlessly to findings from neuropsychological and genetic studies, and have been only moderately successful at predicting treatment outcome (Insel et al. 2010; Retz and Retz-Junginger 2014; Ostacher et al. 2015). Future studies should build on the results from this and similar studies to understand the relationship between behaviour, neurophysiology and the genome to identify syndromes based on pathophysiology. This could lead to more objective and precise approaches to diagnosis and prognosis and may eventually result in improved interventions and long-term outcome (Casey et al. 2014).

## Electronic Supplementary Material

Below is the link to the electronic supplementary material.

Supplementary material 1 (DOCX 12 kb)
