# Supplementary material for: Commonalities in EEG Spectral Power Abnormalities Between Women With ADHD and Women With Bipolar Disorder During Rest and Cognitive Performance
Source: Brain Topogr. 2016 Jul 27;29(6):856–66. doi: 10.1007/s10548-016-0508-0 (PMC5054048; doi:10.1007/s10548-016-0508-0)
Supplement: Supplementary file 1 — Supplementary material 1 (DOCX 12 kb) [file 10548_2016_508_MOESM1_ESM.docx]

***Supplementary material 1***

***Relative EEG power***

The repeated-measure ANOVA indicated no significant main effects of group for relative delta (F_2,57_=0.32, p=0.731), beta 1 (F_2,57_=1.07, p=0.351) or beta 2 (F_2,57_=0.15, p=0.858) power. A significant main effect of group emerged for relative theta (F_2,57_=4.26, p=0.019) and alpha (F_2,57_=3.49, p=0.037) power.

Post-hoc tests revealed significantly reduced relative theta power in the ADHD group compared to controls during both EO (t_38_=-2.86, p=0.007) and CPT-OX (t_38_=-2.08, p=0.045). No significant difference in relative theta power emerged between the BD and control groups during EO (t_38_=-1.97, p=0.057) and CPT-OX (t_38_=-1.58, p=0.123). Furthermore, no significant difference in relative theta power emerged between the ADHD and BD groups during EO (t_38_=0.643, p=0.524) and CPT-OX (t_38_=-1.17, p=0.251). Further post-hoc tests revealed no significant difference in relative alpha power between the ADHD and control groups during EO (t_38_=-1.63, p=0.118) and CPT-OX (t_38_=-1.39, p=0.180). No significant difference in relative alpha power emerged between the BD and control groups during EO (t_38_=-1.73, p=0.093) or CPT-OX (t_38_=-1.95, p=0.059) and between the ADHD and BD groups during EO (t_38_=-1.01, p=0.320) and CPT-OX (t_38_=1.47, p=0.150).

No significant main effects of recording site emerged for relative theta (F_1,57_=2.54, p=0.088; Greenhouse-Geisser ε=0.810), delta (F_1,57_=3.22, p=0.076; Greenhouse-Geisser ε=0.520), alpha (F_1,57_=0.004, p=0.951; Greenhouse-Geisser ε=0.503), beta 1 (F_1,57_=0.257, p=703; Greenhouse-Geisser ε=0.729) or beta 2 (F_1,57_=0.925, p=0.378; Greenhouse-Geisser ε=0.765) power.

There were no significant main effects of testing condition for relative theta (F_1,57_=1.19, p=0.281), delta (F_1,57_=0.37, p=0.545), alpha (F_1,57_=2.85, p=0.097), beta 1 (F_1,57_=0.06, p=0.805) or beta 2 (F_1,57_=0.78, p=0.381) power.

No significant group-by-condition interaction emerged for relative theta (F_1,57_=1.30, p=0.279), delta (F_1,57_=0.11, p=0.899), alpha (F_1,57_=1.77, p=0.179), beta 1 (F_1,57_=0.18, p=0.840) or beta 2 (F_1,57_=0.40, p=0.670) power.

When CPT-OX was segmented based on stimulus-locked epochs (Cue, Go and NoGo), no significant group-by-condition interaction emerged for relative theta (F_1,57_=1.07, p=0.368, Greenhouse-Geisser ε=0.520), delta (F_1,57_=1.40, p=0.217, Greenhouse-Geisser ε=0.376), alpha (F_1,57_=1.07, p=0.382, Greenhouse-Geisser ε=0.822), beta 1 (F_1,57_=0.93, p=0.422, Greenhouse-Geisser ε=0.444) or beta 2 (F_1,57_=0.35, p=0.618, Greenhouse-Geisser ε=0.521) power.

While absolute theta power is increased in ADHD and BD participants compared to controls during the resting-state condition and therefore may indicate commonalities in brain dysfunction between ADHD and BD, relative theta power may act as a marker of neurobiological processes in ADHD only since here we show reduced relative theta power in the ADHD group compared to both controls and BD participants during resting-state and task conditions.
